# Single-Cell RNA Sequencing Efficiently Predicts Transcription Factor Targets in Plants

**DOI:** 10.3389/fpls.2020.603302

**Published:** 2020-12-08

**Authors:** Yunjie Xie, Shenfei Jiang, Lele Li, Xiangzhen Yu, Yupeng Wang, Cuiqin Luo, Qiuhua Cai, Wei He, Hongguang Xie, Yanmei Zheng, Huaan Xie, Jianfu Zhang

**Affiliations:** ^1^College of Plant Protection, Fujian Agriculture and Forestry University, Fuzhou, China; ^2^Rice Research Institute, Fujian Academy of Agricultural Sciences, Fuzhou, China; ^3^State Key Laboratory of Ecological Pest Control for Fujian and Taiwan Crops, Fuzhou, China; ^4^Key Laboratory of Germplasm Innovation and Molecular Breeding of Hybrid Rice for South China, Ministry of Agriculture and Rural Affairs, Fuzhou, China; ^5^Incubator of National Key Laboratory of Germplasm Innovation and Molecular Breeding Between Fujian and Ministry of Sciences and Technology, Fuzhou, China; ^6^Fuzhou Branch, National Rice Improvement Center of China, Fuzhou, China; ^7^Fujian Engineering Laboratory of Crop Molecular Breeding, Fuzhou, China; ^8^Base of South China, State Key Laboratory of Hybrid Rice, Fuzhou, China; ^9^College of Agronomy, Fujian Agriculture and Forestry University, Fuzhou, China

**Keywords:** transcription factor, TF targets, OsNAC78, expression trajectory, scRNA-seq

## Abstract

Discovering transcription factor (TF) targets is necessary for the study of regulatory pathways, but it is hampered in plants by the lack of highly efficient predictive technology. This study is the first to establish a simple system for predicting TF targets in rice (*Oryza sativa*) leaf cells based on 10 × Genomics’ single-cell RNA sequencing method. We effectively utilized the transient expression system to create the differential expression of a TF (OsNAC78) in each cell and sequenced all single cell transcriptomes. In total, 35 candidate targets having strong correlations with OsNAC78 expression were captured using expression profiles. Likewise, 78 potential differentially expressed genes were identified between clusters having the lowest and highest expression levels of OsNAC78. A gene overlapping analysis identified 19 genes as final candidate targets, and various assays indicated that Os01g0934800 and Os01g0949900 were OsNAC78 targets. Additionally, the cell profiles showed extremely similar expression trajectories between OsNAC78 and the two targets. The data presented here provide a high-resolution insight into predicting TF targets and offer a new application for single-cell RNA sequencing in plants.

## Introduction

DNA-binding proteins mediate gene regulation and are involved in most cellular process. In particular, transcription factors (TFs) recognize DNA-binding sites in promoters to modulate gene expression (Nelson, 1995; Luscombe et al., 2000; Ptashne, 2005). Under both normal and stress conditions, thousands of targets are controlled by TFs to maintain intracellular stability. In plants, numerous TFs still have unknown targets, which seriously affects the elucidation of transcriptional networks. At present, several approaches are being used to identify TF targets. One of the most effective approaches is chromatin immunoprecipitation sequencing (ChIP-seq), which has been widely used in animals and plants to identify DNA-binding proteins *in vivo* (Valouev et al., 2008; Barski and Zhao, 2009; Kaufmann et al., 2010). In principle, ChIP-seq relies on specific antigen–antibody recognition to precipitate DNA fragments bound by TFs. These DNA fragments are then isolated and identified to draw conclusions regarding TF transcriptional processes. Although the principle is simple, the technique is challenging, involving many experimental steps and requiring pre-optimization, and it is highly prone to operational errors and artifacts (Perna and Alberi, 2019; Yang et al., 2019). Another technique, microarray-based chromatin immunoprecipitation, requiring a specific antibody, is employed to identify TF targets, but often no significant DNA fragment enrichment is obtained (Mukherjee et al., 2004). Consequently, many researchers ultimately choose bulk RNA-sequencing (RNA-seq) as the simplest approach to predict TF targets through a transcriptome-wide analysis of differentially expressed genes (DEGs; Stark et al., 2019). For RNA-seq, millions of cells are processed in batches that may harbor potential differences, resulting in the generation of a large number of DEGs (Olsen and Baryawno, 2018). It is, however, difficult to subsequently verify the DNA-protein interactions. Thus, there are still many obstacles in using these technique to discover TF targets.

Recently, single-cell RNA sequencing (scRNA-seq) has been applied to various fields. Gene expression profiles are refined to the single-cell level, and unique changes among cell types in a populations are identified at a high resolution (Kolodziejczyk et al., 2015; Macosko et al., 2015; Olsen and Baryawno, 2018). ScRNA-seq has developed into a powerful tool for the analysis of cellular heterogeneity and the discovery of novel cell types in animals. The spatial distribution and pseudotime of numerous individual cells reveal dynamic cell differentiation and developmental trajectories (Han et al., 2018; Papalexi and Satija, 2018). However, its application in plants encountered challenges, in part because of the existence of the cell wall, which hinders the dissociation of tissues into single cells. Fortunately, a series of recent studies have successfully applied scRNA-seq to *Arabidopsis thaliana* roots. A continuous differentiation trajectory in roots and regulators of cell fate determination were revealed across pseudo-time. Simultaneously, transcriptional profiles in heterogeneous cell populations were observed from a high-resolution perspective, even under abiotic stress conditions (Denyer et al., 2019; Jean-Baptiste et al., 2019; Ryu et al., 2019; Zhang T.Q. et al., 2019).

Owing to the emergence of scRNA-seq and its successful application in plants, we hypothesized that this technology could be applied to predict TF targets. Here, we performed high-throughput scRNA-seq to determine expression profiles of rice (*Oryza sativa*) leaf cells. Based on differential expression levels of the NAC (NAM, ATAF, and CUC) TF OsNAC78 in cells, potential expression characteristics were used to correlate the TF with its targets. Using DNA-binding assays for verification, we finally identified two targets of OsNAC78. Thus, this study provides a rapid and highly efficient approach to predict TF targets.

## Materials and Methods

### Protoplast Isolation, Transfection, and Western Blotting

Rice protoplast isolations were performed as described previously (Zhang et al., 2011). Briefly, the indica cultivar *MH63* rice seedlings that had been grown for 3 weeks were selected as the source material. The leaves of the rice seedlings were cut and immediately transferred into the enzyme solution (1.5% cellulase R-10, 0.75% macerozyme R-10, 0.6 M mannitol, and 10 mM MES at pH 5.7). They were mixed with the enzyme solution for 3 h at 28°C and 70 rpm in the dark. After pouring off the enzyme solution, the W5 solution (154 mM NaCl, 125 mM CaCl_2_, 5 mM KCl and 2 mM MES at pH 5.7) was added and shaken vigorously to release the protoplasts, which were filtered through a 250-micron mesh and two layers of nylon mesh. The protoplasts were collected by centrifugation at 300 × *g*, washed with the W5 solution after which the supernatant was removed by centrifugation. The protoplasts were resuspended using MMg solution (0.6 M D-Mannitol, 15 mM MgCl_2_ and 4 mM MES at pH 5.7). Each sample consisting of 5 μg green fluorescent protein (GFP)-containing plasmids and 100 μL rice protoplasts was transfected using the PEG-mediated method as described previously (Yoo et al., 2007). Samples were cultured for 4, 5, 8, and 12 h at 28°C in the dark, and then, the fluorescence of transfected cells was observed using a confocal microscope (Leica, Germany). The pRTVcOsNAC78-HA and pRTVcHA plasmids were independently transfected using the above methods and then cultured for 8 h at 28°C in the dark. The cell concentration and cell viability were determined using a hemocytometer and Trypan Blue staining, respectively, and then 10 × Genomics’ scRNA-seq was performed. To confirm that the OsNAC78-HA plasmids were successfully transfected into the protoplasts, the total protein contents of transfected protoplasts were extracted, and western blotting assays were performed using anti-HA, as described previously (Zhang et al., 2011).

### ScRNA-Seq

The scRNA-seq libraries were prepared using Chromium Single cell 3′ Reagent v2 Kits in accordance with the manufacturer’s protocol. Single cells were loaded onto the Chromium Single Cell Controller Instrument (10× Genomics) to generate single cell gel beads in emulsions (GEMs). After the generation of single cell GEMs, reverse-transcription reactions were performed to produce barcoded full-length cDNAs, followed by the disruption of GEMs using the recovery agent and cDNA clean up with DynaBeads Myone Silane Beads (Thermo Fisher Scientific). cDNAs were then amplified by PCR using 12 cycles. Subsequently, the amplified cDNAs were fragmented, end-repaired, A-tailed and index adaptor-ligated. The resulting libraries were amplified. Then, these libraries were sequenced on the Illumina sequencing platform (NovaSeq 6000), and 150-bp paired-end reads were generated.

### Preprocessing and Analyzing ScRNA-Seq Data

Raw sequencing datasets were analyzed using the Cell Ranger software pipeline (version 2.2.0) provided by 10× Genomics. The pipeline included demultiplexing cellular barcodes, generating map reads to the genome and transcriptome using STAR aligner, and creating down-sample reads as required to generate normalized aggregate data across samples, which produced a matrix of gene counts versus cells. The gene–cell matrices for samples were further processed using the R package Seurat (version 2.3.4). To remove low-quality cells and doublets, we filtered out cells with unique molecular identifiers (UMIs)/gene numbers outside of the mean value +/- two fold of the standard deviation limit, assuming a Gaussian distribution of each cells’ UMI/gene number (Jean-Baptiste et al., 2019). Following visual inspection of the distribution of cells based on the fraction of mitochondrial genes expressed, we further discarded low-quality cells where >10% of the counts belonged to mitochondrial genes. After applying these QC criteria, 5,912 genes across 8,260 cells were used for further analyses in transiently over-expressed OsNAC78 samples (TOE). In total, 7,722 genes across 16,527 cells were used for further analyses in controls and TOEs.

Expression datasets were first normalized using the LogNormalize method, and 500 DEGs were detected with FindVariableFeatures function. After scaling using ScaleData, a principle component analysis (PCA) was performed using RunPCA with 100 potential PCs. Clusters were identified using FindClusters at a resolution of 1.4. The final data structure and cell clusters were visualized after the application of the RunTSNE function. Marker genes for each cluster were identified using FindMarkers, with the minimum cell percentage for a marker gene being more than 25% and the fold change of the average expression levels between two clusters being more than 1.5 (Jean-Baptiste et al., 2019). The expression levels of genes were plotted using the FeaturePlot function. To evaluate the correlations between OsNAC78 and candidate target genes, mean normalized expression levels for each cluster were calculated and Pearson’s correlation coefficients were calculated using R v3.6.1.

To understand the basic roles of detected genes in our sample, gene ontology (GO) enrichment analysis was performed using topGO. A GO term with a *p*-value < 0.05 was regarded as significantly enriched.

### Construction of the Expression Vector and Rice Transformation

The coding region of OsNAC78 was amplified using specific primers containing the restriction sites *Pst I* and *BamH I*. The resulting OsNAC78 fragment was inserted into the *Pst I* and *BamH I* site of pCUbi1390, generating Ubipro::OsNAC78. The vector was introduced into *Agrobacterium tumefaciens* stain *EHA105*, and then transferred into embryogenic calli of *Nipponbare* via *Agrobacterium*-mediated transformation. T2 seeds were obtained for further research.

### Quantitative Real-Time PCR (qRT-PCR)

To investigate the expression levels of candidate genes in OsNAC78-overexpression lines, we extracted total RNAs from rice leaves using TRIzol reagent (TransGen Biotech, China). The total RNA was reverse transcribed into cDNA using a RNA reverse-transcription kit with gDNA Remover (Toyobo, Japan) in accordance with the manufacturer’s instructions. qRT-PCR was performed using a FastStart Universal SYBR Green Master (ROX) Kit (Roche, United States) on an ABI Prism^®^ 7500 Real-time PCR system. The reaction solution contained 10 μL SYBR Green Mix, 8.25 μL aseptic water, 0.375 μL each primer and 1 μL cDNA. The thermal cycling conditions were as follows: 95°C for 10 min, 40 cycles of 95°C for 10 s and 60°C for 30 s. The *ubiquitin* gene was used as an internal control, and the relative expression levels were obtained using the ΔΔCt method. All the experiments were repeated biologically three times. All the primes are listed in Supplementary Table 1.

### Yeast One-Hybrid Assay

The yeast one-hybrid assay was performed using the vector pLacZi or pJG4-5 and the yeast strain EGY48. The full-length CDS of OsNAC78 was amplified using specific primers and inserted into vector pJG4-5. The promoter fragments of candidate genes were amplified using the appropriate corresponding primers and inserted into the pLacZi vector. These constructed vectors and the pJG4-5-OsNAC78 vector were co-transformed into the EGY48 yeast strain using the PEG/LiAc method. The transformed yeast cells were grown on SD/-Trp/-Ura medium and then applied to the yeast plates containing 5-bromo-4-chloro-3-indolyl β-D-galactoside (X-Gal). Interactions were screened by the presence of a blue pigment. The empty vector pJG4-5 and recombinant pLacZi vectors were co-transformed as negative controls.

### Electrophoretic Mobility Shift Assay (EMSA)

The CDS of OsNAC78 was inserted into the pMAL-c5x vector. The maltose-binding protein (MBP) and MBP-OsNAC78 fusion proteins were express in *Escherichia coli* (Rosetta) and purified. The double-stranded Cy5.5-labeled probes used in this assay were synthesized (Biosun, China), and the sequences are listed in Supplementary Table 1. The EMSA assay was performed using an EMSA/Gel-Shift Kit (Beyotime, China) following the manufacturer’s instructions. Briefly, 2 mg of purified MBP or MBP-OsNAC78 protein was added to the binding reaction and incubated for 20 min at 25°C in a thermal cycler (Bio-Rad, United States). The mixture was separated on a 4% polyacrylamide gel in 0.5× Tris-Borate-EDTA buffer, and the gel images were taken using the Odyssey^®^ Infrared Imaging System (LI-COR, United States).

### Dual-Luciferase (Dual-LUC) Transcriptional Activation Assay

To analyze transcriptional activity, we used a dual-LUC reporter assay system in rice protoplasts. First, the promoters of Os01g0934800 and Os01g0949900 were inserted into the LUC reporter vector pGreen II 0800, which included a Renilla LUC (REN) gene driven by CaMV 35S as an internal control. Then, the pRTVcOsNAC78-HA vector acted as an effector. The reporter and effector plasmids were co-transformed into protoplasts using the above described method. The transformed protoplasts were cultured for 36 h at 28°C in the dark, and the activities of LUC and REN were measured in accordance with the Dual Luciferase Reporter Assay Kit’s instructions (Vazyme, China). The min35s promoter was used as a negative control. The binding capacities of OsNAC78 to the candidate genes’ promoters were expressed as LUC/REN ratios. All the experiments were repeated biologically three times.

## Results

### Workflow of Predicting TF Targets Based on 10× Genomics’ ScRNA-Seq

**FIGURE 1:**
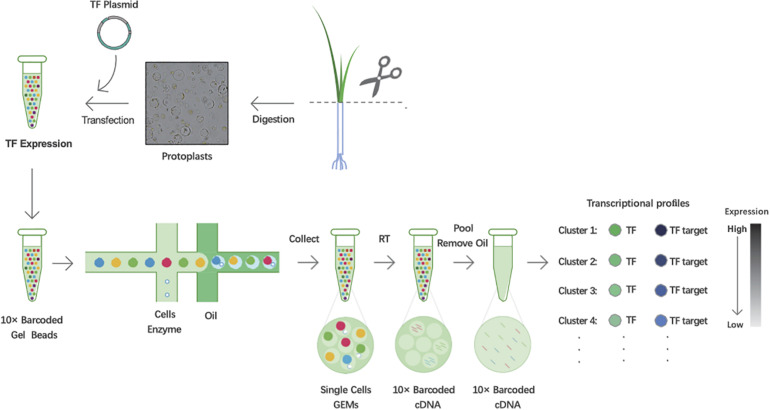
Workflow schematic of predicting transcription factor (TF) targets in rice using single-cell RNA sequencing (scRNA-seq). Scissor:cut the leaves together into approximately 0.5 mm stipes.

To predict TF-binding targets using 10× Genomics’ scRNA-seq, leaves of 3-week-old rice seedlings were harvested and digested to obtain protoplasts. Using the PEG-mediated transfection method, the TF plasmids were introduced into protoplasts for expression. Then, cDNA fragments in each single cell were labeled using 10× Genomics’ barcoded gel beads, and the gel beads containing barcode information bound to a mixture of cells and enzymes encapsulated by oil surfactant droplets located in the microfluidic “double cross” connection. Then, the droplets flowed into a pool and were collected. Using the barcode information, the sequencing libraries were constructed. Finally, based on the expression correlations between TF and targets, we utilized transient expression differences in TFs in each cell to capture the associated potential targets (Figure 1).

### Sample Preparation and 10× Genomics’ ScRNA-Seq

**FIGURE 2:**
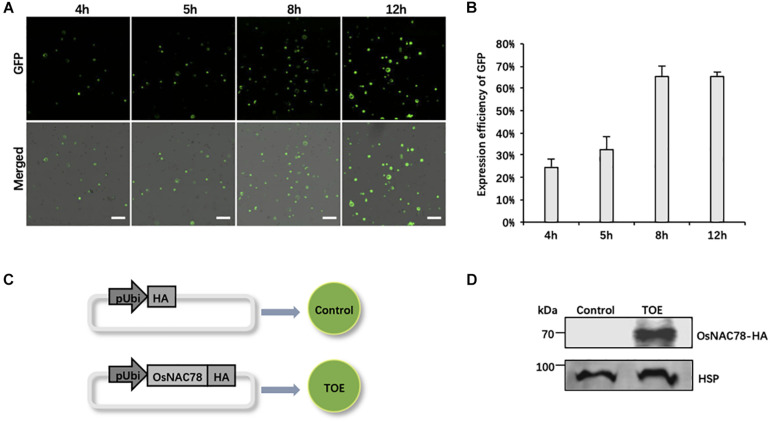
The preparation of rice scRNA-seq samples. **(A)** Confocal micrographs showing the GFP signals at different times after transfecting rice protoplasts with the pRTVcGFP vector. Scale bar, 200 μm. **(B)** Histogram showing the expression efficiency of GFP at different times after transfection into rice protoplasts. Data are presented as means ± *SD* (*n* = 3). **(C)** Schematic representation of the treatment of rice scRNA-seq samples. Protoplasts transfected with the pRTVcHA vector were used as controls. Protoplasts transfected with the pRTVcOsNAC78-HA vector were used as transiently over-expressed OsNAC78 samples (TOE). pUbi, *ubiquitin* promoter. HA, HA-tag protein. The circles denote rice leaf protoplasts. **(D)** The protein expression levels of OsNAC78-HA in the control and TOE were detected by an anti-HA polyclonal antibody using western blotting. HSP, which served as an internal control, was detected using an anti-HSP polyclonal antibody.

Owing to the efficiency of the PEG-mediated transient expression, it is necessary to optimize the experimental conditions. We used the empty vector pRTVcGFP carrying the *ubiquitin* (Ubi) promoter to drive GFP as a reference, and the expression efficiency of GFP at different periods after transfection was observed. The confocal observations showed that GFP signals began to sporadically appear at 4 h and then gradually increased. The expression efficiency and level both peaked at 12 h (∼65% of cells fluoresced). Notably, the expression efficiency of GFP was relatively complete, and cells had the most diverse GFP expression levels at 8 h (Figures 2A,B). Thus, we finally chose 8 h after transfection as the best time to collect samples.

We used the TF gene Os02g0822400 (OsNAC78) as our research object, and scRNA-seq as a tool to identify targets of OsNAC78. The TOE transfected with the pRTVcOsNAC78-HA vector, having the HA tag at the C-terminus, were used as the experimental group, and cells transfected with pRTVcHA were used as the control (Figure 2C). Samples were collected after transfection, and single-cell suspensions for each sample were examined, with more than 1,000 cells per μL present. Cell survival rates were more than 85%, which met the standard for sequencing (Supplementary Table 2). Additionally, the OsNAC78-HA protein in the TOE was detected by western blotting, indicating that OsNAC78-HA was overexpressed in cells, but it was not detected in the control (Figure 2D). Therefore, we successfully obtained qualified samples and then performed scRNA-seq.

### Predicting OsNAC78 Targets Using a ScRNA-Seq Analysis

The transcriptome of rice leaf cells was profiled using the 10 × Genomics’ platform. In total, 12,891 and 13,639 cells were captured in the control and TOE, respectively, containing means of 5,495 and 5,072 UMIs per cell, respectively. In total, 24,715 and 24,801 genes were detected in the control and TOE, respectively, with means of 1,913 and 1,765 genes, respectively, detected per cell (Supplementary Table 3). After quality filtering, some individual cells having gene/UMI numbers that exceeded the thresholds were discarded, and a mean of 2,050 genes was ultimately detected among 16,527 cells (Supplementary Figure 1). A total of 12,842 genes were detected for further analysis. To understand the basic information in our experiments, GO enrichment analysis was firstly performed (Supplementary Figure 2). Genes involved in basic ontology terms were enriched, such as cellular process, catabolic process, biosynthetic process and reproduction. Genes with binding function and structural molecule activity were detected in our analysis. In cellular component terms, genes located in nucleolus, cytosol, mitochondrion or membrane region were enriched in our dataset.

**FIGURE 3:**
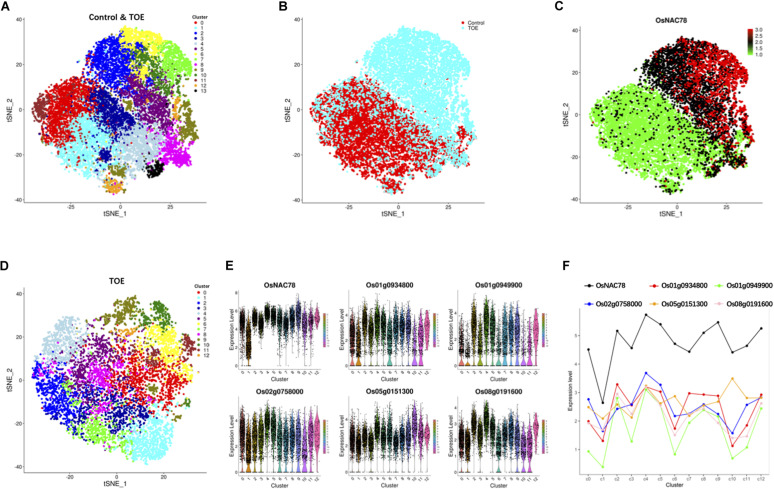
Cluster and correlation analysis of rice leaf cells. **(A)** t-SNE visualization identifying 14 clusters in the control and TOE. Each dot denotes a single cell. **(B)** t-SNE visualization identifying the profiles of control cells and TOE cells. Each dot denotes a single cell. **(C)** t-SNE visualization of OsNAC78’s expression in the control and TOE. Each dot denotes a single cell. The depth of color indicates differences in the expression levels. **(D)** t-SNE visualization identifying 13 clusters in the TOE. Each cluster contained from 74 to 1,077 cells. Each dot denotes a single cell. **(E)** Violin plot showing the expression levels of OsNAC78 and 5 of the 65 candidate genes whose expression correlated with that of OsNAC78 in the TOE. Each dot denotes a single cell. **(F)** Line chart showing the average expression levels of OsNAC78 and 5 of the 65 candidate genes whose expression correlated with that of OsNAC78 in each cluster of the TOE. A dot represents the mean value of a cluster.

Following gene expression normalization for read depth, analysis PCA was performed using the top 500 highly variable expressed genes. An unsupervised clustering with a t-distributed stochastic neighbor embedding (t-SNE) analysis was performed on the transcriptomes using the Seurat software package to visualize and explore the datasets (van der Maaten and Hinton, 2008). The leaf cells in the control and TOE were grouped into 14 cell clusters on the basis of marker genes in the cell populations (Figure 3A). Interestingly, two district expression profiles were identified in the control and TOE, suggesting that OsNAC78 had effects on the TOE cells (Figure 3B). Compared with the control, the expression of OsNAC78 was significantly induced in the TOE (Figure 3C). The results indicate that OsNAC78 was successfully expressed and stimulated related gene expression levels in the TOE cells. In this t-SNE analysis, we obtained closely arranged clusters, derived from the low heterogeneity of rice leaf cells (Figures 3A–C). This indicated that single cell trancriptomes were almost identical, ensuring that the downstream transcriptional targets regulated by NAC78 were screened under the condition of relatively homogeneous transcriptomes of individual cell.

**FIGURE 4:**
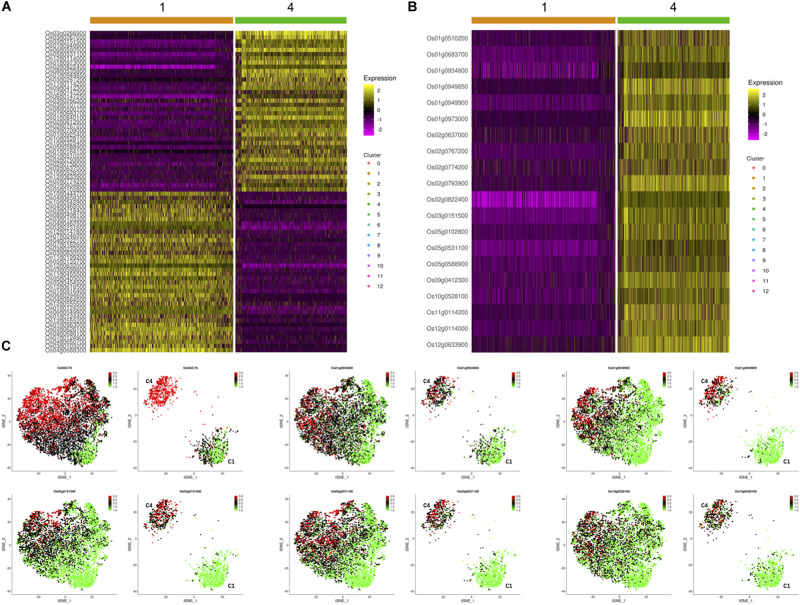
The expression profiles of OsNAC78’s candidate targets in the TOE. **(A)** Heat map illustrating the transcript levels of 78 DEGs between clusters 1 and 4, which had the lowest and highest OsNAC78 expression levels, respectively. Os02g0822400, OsNAC78. The bars at the top of the heat map indicate the clusters in the TOE. **(B)** Heat map illustrating the transcript levels of OsNAC78 and 19 overlapping genes generated from 35 strongly correlated genes and 78 DEGs between clusters 1 and 4. Os02g0822400, OsNAC78. The bars at the top of the heat map indicate the clusters in the TOE. **(C)** t-SNE visualization of OsNAC78 and 5 of the 19 overlapping genes in the TOE and isolated from cluster 1 or 4. C1, cluster 1; C4, cluster 4. Each dot denotes a single cell. The depth of color indicates differences in expression levels.

To investigate the effects of OsNAC78 expression on leaf cells, 13 clusters, each containing from 74 to 1,077 cells, were identified using t-SNE visualization based on the expression profile of the TOE cells (Figure 3D). A heat map illustrated the transcript accumulations of the top 10 marker genes in each cluster (Supplementary Figure 3). Cluster-specific marker genes were identified on the basis of their expression levels in each cluster compared with all the other clusters (fold-change > 1.5 and false discovery rate <0.05; Supplementary Table 4). OsNAC78 had diverse expression levels among the clusters, especially in clusters 1 and 4 (Figures 3E,F). On this basis, the expression levels of 65 candidate genes were correlated with the expression of OsNAC78 (Figures 3E,F and Supplementary Figure 4). The average expression levels of the 65 candidate genes among the 13 clusters were calculated, and strong correlations for 35 candidate genes were observed (Pearson’s correlation coefficient R^2^ > 0.7; Supplementary Table 5). Furthermore, DEGs were identified between cluster 1, having the lowest OsNAC78 expression level, and cluster 4, having the highest OsNAC78 expression level (Figures 3E,F). In total, 265 genes changed their expression levels between cells in clusters 1 and 4 (fold-change > 1.5 and false discovery rate <0.05; Supplementary Table 6), with 128 genes, including OsNAC78, significantly induced in cluster 4 and 78 DEGs expressing two-fold higher than in cluster 1 (Figure 4A).

Using a gene overlapping analysis, 19 candidate genes were identified from the 35 strongly correlated genes and 78 DEGs (Supplementary Table 7). A heat map of all 19 overlapping genes in clusters 1 and 4 were constructed and their expression profiles in the TOE determined (Figures 4B,C and Supplementary Figure 5). We clearly observed that the expression patterns of these 19 genes were similar to that of OsNAC78. Thus, all 19 genes were selected as final candidate target genes of OsNAC78 and subjected to further experimental validation.

### OsNAC78 Directly Binds to the Promoters of Os01g0934800 and Os01g0949900

**FIGURE 5:**
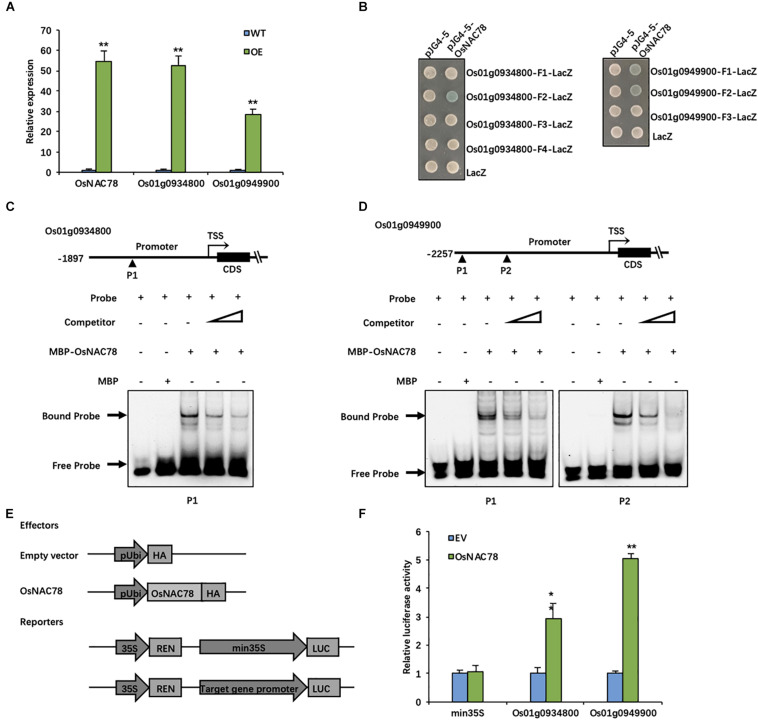
OsNAC78 directly binds to the promoters of Os01g0934800 and Os01g0949900. **(A)** The relative expression levels of OsNAC78, Os01g0934800, and Os01g0949900 were detected in wild-type (WT) and OsNAC78 overexpression (OE) lines as assessed by qRT-PCR. Data are presented as means ± *SD*s (*n* = 3) and were analyzed using Student’s *t*-test; The asterisks indicate significant differences (**P < 0.01). **(B)** The interactions between OsNAC78 and the promoter fragments of Os01g0934800 (represented as F1–4) and Os01g0949900 (represented as F1–3) were shown using a yeast one-hybrid assay. Yeast cells were grown on SD/-Ura/-Trp–X-gal medium. pJG4-5 was used as an effector vector. pLacZi (LacZ) was used as a reporter vector. **(C)** The interaction between OsNAC78 and a probe (P1) containing one CGTG motif in the Os01g0934800 promoter was shown using EMSA. Unlabeled probe was used as a competitor. The triangle denotes an increasing dose. MBP was used as a control. **(D)** The interaction between OsNAC78 and a probe (P1) containing two CGTG motifs or a probe (P2) containing one CGTG motif in the Os01g0949900 promoter was shown using EMSA. Unlabeled probe was used as a competitor. The triangle denotes an increasing dose. MBP was used as a control. **(E)** Schematic showing the vectors used for identifying interactions between OsNAC78 and the promoters of Os01g0934800 and Os01g0949900 using a dual-luciferase (LUC) assay. OsNAC78 was inserted into the pRTVcHA vector as an effector. The promoters of Os01g0934800 and Os01g0949900 were independently inserted into the pGreenII 0800-LUC vector as reporters, and Renilla luciferase (REN) was used as an internal control. **(F)** The relative LUC activity levels indicated the promoter activities of min35S, Os01g0934800 and Os01g0949900 in the absence and presence of OsNAC78 in protoplasts. The min35S promoter was used as a control. EV, empty vector. Data are presented as means ± *SD*s (*n* = 3) and were analyzed using Student’s *t*-test; The asterisks indicate significant differences (**P < 0.01). *P < 0.05.

To conduct efficient validations of the 19 candidate genes, we generated transgenic lines that overexpressed OsNAC78, and then, we assessed the expression levels of the candidate genes using qRT-PCR. The CDS of OsNAC78 was inserted into the pCUbi1390 vector and transformed into the *Nipponbare* background. Os01g0934800 and Os01g0949900 were both significantly up-regulated when OsNAC78 was overexpressed (Figure 5A). Seven and eight CGT[GA] motifs in the promoter regions of Os01g0934800 and Os01g0949900, respectively, were identified by a sequence analysis. These elements are likely to be DNA-binding sites of OsNAC78, because NAC TFs recognize CGT[GA] core motifs (Tran et al., 2004; Jensen et al., 2010; Jensen and Skriver, 2014).

Thus, a yeast one-hybrid assay was used to determine the direct binding between OsNAC78 and the promoters of Os01g0934800 and Os01g0949900. OsNAC78 directly bound to the F2 (−1,395—1,116) promoter region of Os01g0934800 and to the F1 (−2,251—1,992) and F2 (−1,643—1,587) promoter regions of Os01g0949900 (Figure 5B). All three promoter fragments contained CGTG motifs. To further determine whether OsNAC78 specifically binds to these motifs, an EMSA was performed. The double-stranded Cy5.5-labeled probes used in this assay were synthesized based on these motif regions, and unlabeled probes were used as competitors. The OsNAC78 protein fused to an N-terminal MBP tag was successfully expressed in *E. coli* (Rosetta). MBP-OsNAC78 bound labeled probes containing CGTG motifs to form DNA–protein complexes. Then, migration was attenuated by increasing doses of competitive probes (Figures 5C,D). Our results indicated that OsNAC78 directly bound the CGTG motifs in promoter fragments of Os01g0934800 and Os01g0949900.

The scRNA-seq results indicated that OsNAC78 is positively correlated with Os01g0934800 and Os01g0949900. However, this did not prove that it acts as an activator. We performed a dual-LUC assay to determine the correlations between OsNAC78 and both Os01g0934800 and Os01g0949900. The OsNAC78-HA fusion protein driven by the *ubiquitin* promoter and the Firefly LUC driven by the target gene promoter acted as an effector and a reporter, respectively (Figure 5E), when co-transfected into rice protoplasts. The LUC transcriptional activity driven by the target genes’ promoters were remarkably up-regulated in cells transiently over-expressed OsNAC78, demonstrating that OsNAC78 interacted with the two target genes’ promoters, but not with the min35S promoter (Figure 5F). Additionally, OsNAC78 positively regulated the expression levels of Os01g0934800 and Os01g0949900. These observations showed that Os01g0934800 and Os01g0949900 are downstream targets of OsNAC78.

### Analyzing the Expression Profiles of Target Genes Os01g0934800 and Os01g0949900 in the Control and TOE

**FIGURE 6:**
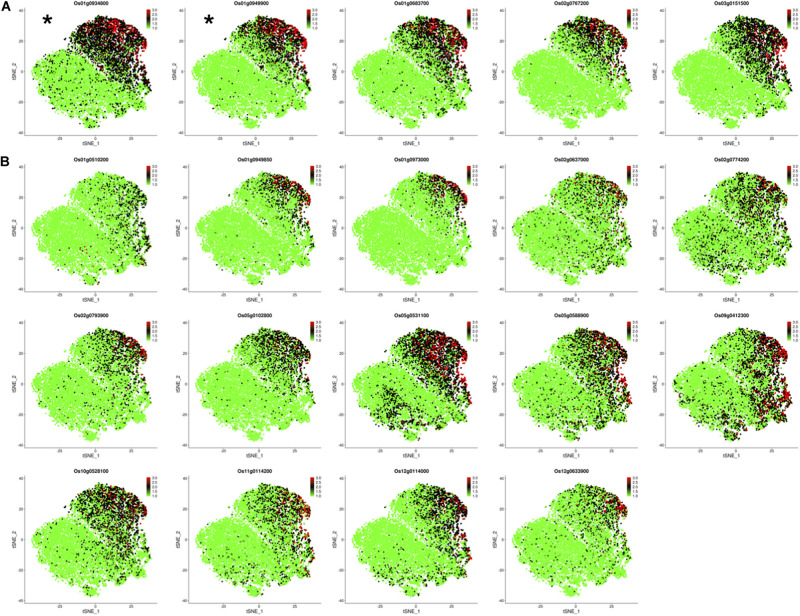
The expression profiles of 19 candidate genes in the control and TOE. **(A)** t-SNE visualization of five candidate genes having expression trajectories that were highly similar to that of OsNAC78. *OsNAC78 target. Each dot denotes a single cell. The depth of color indicates different expression levels. **(B)** t-SNE visualization of 14 candidate genes having expression trajectories that were somewhat similar to that of OsNAC78. Each dot denotes a single cell. The depth of color indicates different expression levels.

Os01g0934800 and Os01g0949900 have been identified as OsNAC78 targets from 19 candidate genes based on experimental results. The expression profiles of the two targets in the control and TOE need to be further studied for the efficient identification of targets. We determined the expression of 19 candidate genes using t-SNE visualization in the control and TOE (Figure 6). Notably, five genes, including targets Os01g0934800 and Os01g0949900, displayed expression trajectories highly similar to that of OsNAC78 (Figures 3C, 6A). While the other 14 genes showed low correlations and different expression trajectories, compare with that of OsNAC78 (Figures 3C, 6B). Although the interaction between OsNAC78 and the 19 candidate genes cannot be all clearly verified owing to experimental limitation, we found that genes having expression trajectories similar to the TF are more likely to be targets. Therefore, the efficiency of capturing targets is improved by screening candidate genes based on transcriptional similarity to the TF at a high resolution.

## Discussion

With the development of droplet technology, scRNA-seq has been applied as a research tool in many fields (Klein et al., 2015; Zhang X.N. et al., 2019). However, it is unusual to identify TF targets using the high-throughput 10 × Genomics’ platform. At present, directly testing the binding between TF and DNA fragments *in vivo* using Chip-seq is preferred. However, its experimental operation is demanding, the steps are cumbersome and error-prone, and the pseudomorphisms are highly sensitive (Perna and Alberi, 2019; Yang et al., 2019). Therefore, bulk RNA-seq, which captures candidate targets using a single dimensional analysis of DEGs, is often used as a simple tool to predict TF targets instead of Chip-seq.

scRNA-seq is an upgrade of RNA-seq that is not limited to DEG analyses, but is a multidimensional analysis, which greatly improves the screening efficiency for identifying TF targets. For example, in the scRNA-seq data presented here, 78 DEGs were identified as candidate targets between clusters having the lowest and highest expression levels of OsNAC78 in the TOE (Figure 4A). The DEGs analysis of one method alone was not able to ensure accurate results. However, taking clusters as units, 35 genes strongly correlated with OsNAC78 were obtained (Supplementary Table 5). In total, 19 overlapping genes were acquired by comparing the two lists, and it was likely that OsNAC78 targets were present. Compared with bulk RNA-seq, scRNA-seq has an additional dimension for scanning the transcriptome, which is an eligible predictor of TF targets.

Furthermore, the superiority of scRNA-seq is also reflected in the reduction of potential errors. In bulk RNA-seq, sampling from different individual plants makes it impossible to avoid identifying false positive genes, because the DEGs are derived from potential differences in growth conditions or external factors. However, scRNA-seq can avoid this error. A separate sample is sufficient for predicting TF targets, such the TOE, in which each cell is treated almost the same during the collection process. Thus, external interference can be basically eliminated, resulting in a reduction in false positive results.

Although scRNA-seq has high-resolution visualization, there are also several factors that affect the data analysis. For example, the TOE was grouped into 13 clusters, taking the average expression of OsNAC78 for correlation analysis on the cluster unit. The greater the number of clusters, the fewer candidate targets with higher precision may be identified in a manner. Unfortunately, when we adjusted the resolution to cell unit for the correlation analysis, we were not able to acquire candidate targets associated with OsNAC78, and the data appeared irregular. There may be two reasons, as follows: (1) some noisy, sparse and high-dimensional cells cannot be eliminated and they interfere with the data analysis (Kulkarni et al., 2019); or (2) scRNA-seq easily captures highly expressed transcripts in cells, while genes with low and medium expression levels are unreliably quantified, including genes having zero reads (Kharchenko et al., 2014; Huang et al., 2018). When performing a correlation analysis on the cell unit, such genes may affect the accuracy. More data algorithms for single-cell expression recovery need to be developed to estimate the actual numbers of transcripts of all the genes. Thus, scRNA-seq still poses challenges when applied to plants.

Using t-SNE visualization, the cell clusters were closely arranged in our profiles (Figures 3A,D). This indicates that cell heterogeneity in rice leaves is low. Cell type-specific transcript abundance gradually decreases as chloroplasts mature, especially in bundle sheath cells and mesophyll cells (Sharpe et al., 2011). During the preparation of our samples, 3-week-old seedlings were chosen as the experimental material, and the rice leaves we investigated were mostly mature, which might explain the low level of cell heterogeneity. Thus, we hypothesize that juvenile leaves may be more suitable for scRNA-seq when studying cell type. Likewise, rice does not currently have as abundant cell-type marker genes as *Arabidopsis*; therefore, it is difficult to distinguish the cell type of each cluster in rice. But in this study, clustering was only used to correlate target genes effectively. The low cell heterogeneity reflects that single cell transcriptomes are almost identical, on which basis the accuracy of associated NAC78 target genes can be improved. In addition, we discovered that the arrangements of the two cell populations in the control and TOE were significantly different, because gene transcription in most TOE cells had altered (Figure 3B). It is possible that the overexpression of OsNAC78 stimulated downstream related gene expression, resulting in two district expression profiles. This indicates the OsNAC78 may play an important role in rice.

OsNAC78 is a member of the NAC family. The NAC TFs have been identified as plant-specific proteins (Nuruzzaman et al., 2010; Welner et al., 2012). There are more than 150 NAC TFs in rice, and they contain a conserved N-terminal DNA-binding domain and a variable C-terminal transcriptional activation region (Ooka et al., 2003; Ernst et al., 2004). NAC TFs play essential roles in biotic and abiotic stress responses (Wu et al., 2009; Nuruzzaman et al., 2013; Sun et al., 2015). However, the function of OsNAC78 has not yet been identified in rice. Two targets, Os01g0934800 and Os01g0949900, were identified in this study. Os01g0934800 encodes esterase Pir7b, a defense-related rice protein, which may have a detoxification role in defense responses against *Pyricularia oryzae* (Luo et al., 2008). Os01g0949900 is a *GST* gene encoding a ROS-scavenging enzyme that may prevent oxidative bursts and inhibit oxidative damage under stress conditions (Wagner et al., 2002; Dean et al., 2005). The functions of these two targets provide clues to the roles of OsNAC78.

## Data Availability Statement

The authors acknowledge that the data presented in this study must be deposited and made publicly available in an acceptable repository, prior to publication. Frontiers cannot accept a manuscript that does not adhere to our open data policies.

## Author Contributions

JZ and HuX planned and designed the experiments. YX, XY, and JZ wrote the manuscript. YX, SJ, LL, and JZ performed the experiments and analyzed the data. YW, CL, QC, WH, HoX, and YZ were in charge of collection and interpretation some materials. All authors contributed to the article and approved the submitted version.

## Conflict of Interest

The authors declare that the research was conducted in the absence of any commercial or financial relationships that could be construed as a potential conflict of interest.

## Supplementary Material

Supplementary Figure 1 Each cells’ gene/UMI numbers before or after quality filtering.

Supplementary Figure 2 Gene ontology analysis of detected genes in control and TOE sample.

Supplementary Figure 3 The transcript levels of top 10 marker genes in each cluster of the TOE.

Supplementary Figure 4 The expression levels of 60 of the 65 candidate genes in the TOE.

Supplementary Figure 5 The expression profiles of 14 of the 19 overlapping genes in the TOE and isolated from cluster 1 or 4.

Supplementary Table 1 Primers list.

Supplementary Table 2 The index of samples used for 10× Genomics’ scRNA-seq.

Supplementary Table 3 The statistical quality results of sequencing date.

Supplementary Table 4 Cluster-specific marker genes in the TOE.

Supplementary Table 5 65 correlation genes in the TOE.

Supplementary Table 6 265 DEGs between cluster 1 and 4 in the TOE.

Supplementary Table 7 19 overlapping genes.
