# Supplementary material for: Single-Cell RNA Sequencing Efficiently Predicts Transcription Factor Targets in Plants
Source: Front Plant Sci. 2020 Dec 8;11:603302. doi: 10.3389/fpls.2020.603302 (PMC7793804; doi:10.3389/fpls.2020.603302)
Supplement: Supplementary Figure 1 — Each cells’ gene/UMI numbers before or after quality filtering. [file Data_Sheet_2.PDF]

Supplementary figures

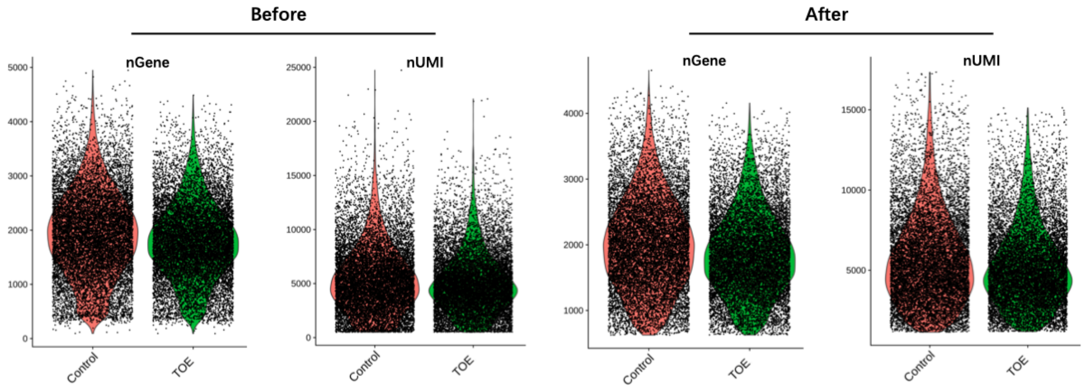

Supplementary Figure S1. Violin plot showing each cells' gene/UMI numbers in scRNA-seq samples before or after quality filtering. Each dot denotes a single cell.

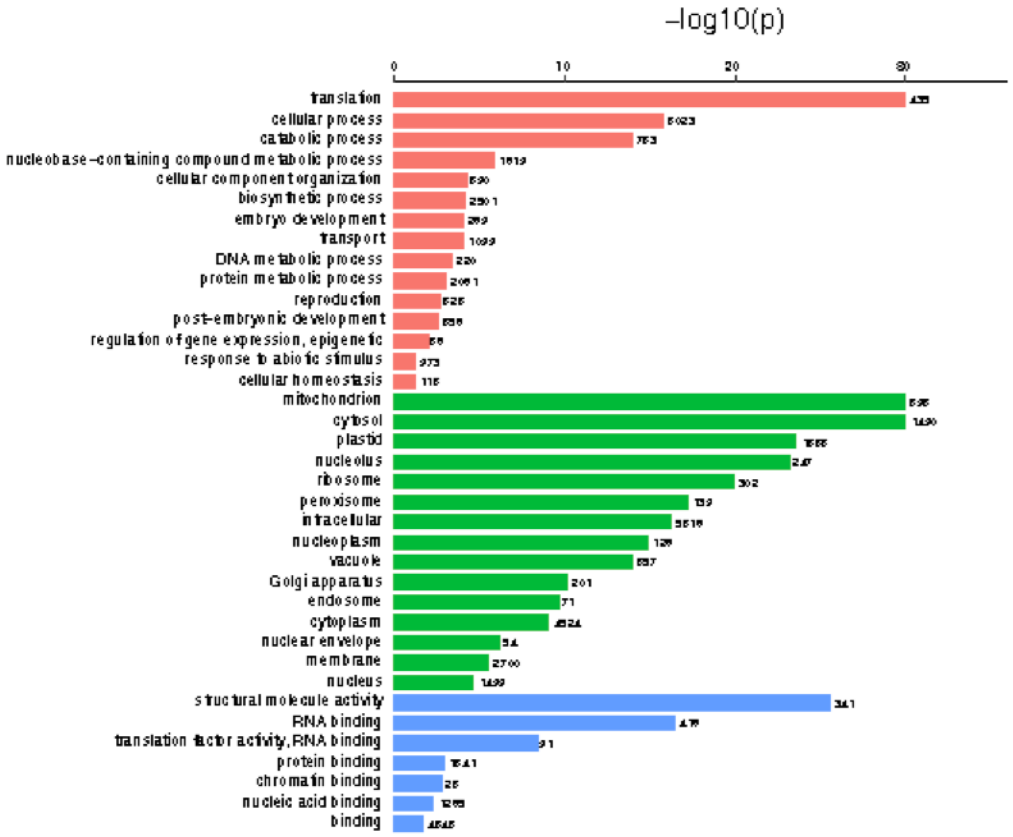

Supplementary Figure S2. Gene ontology analysis of detected genes in control and TOE sample. A GO term with a p-value < 0.05 was regarded

as significantly enriched. Gene ontology terms in bilogical process(red), cellular component(green) and molecular function(blue) were ploted.

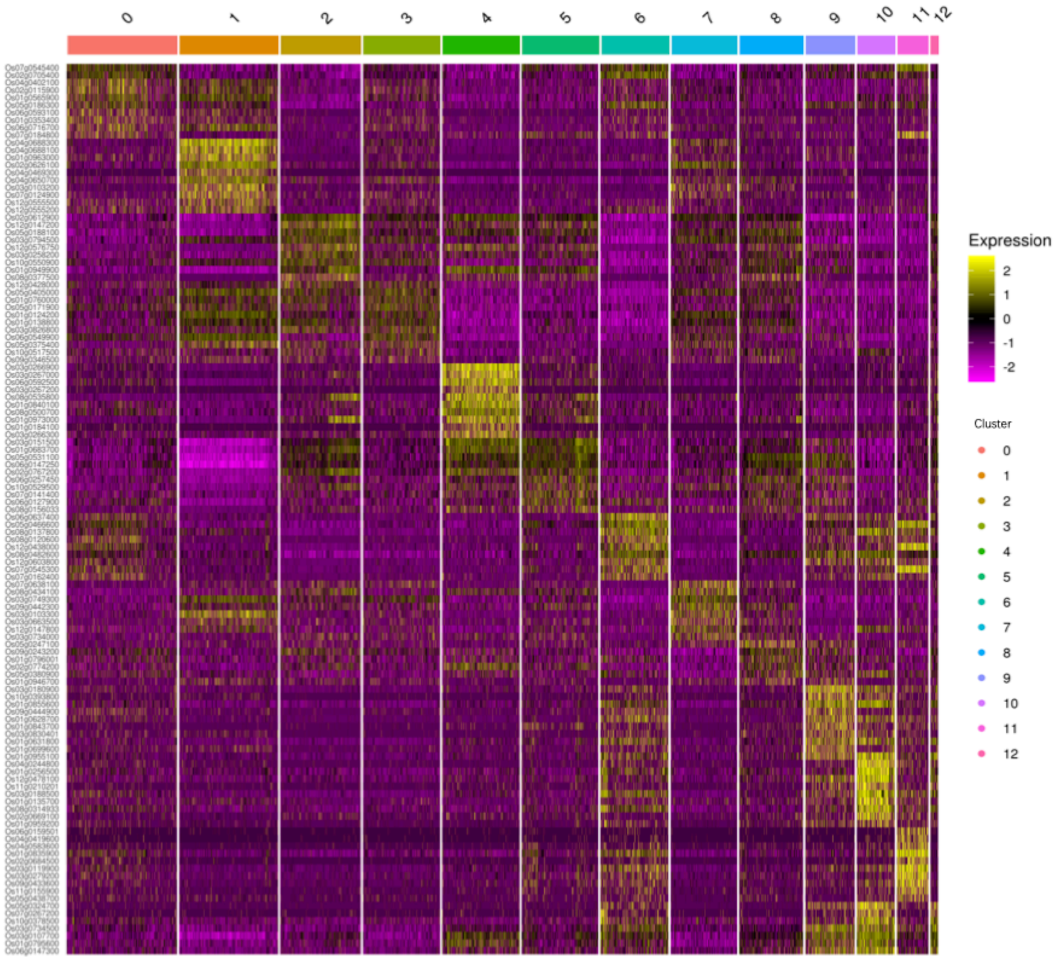

Supplementary Figure S3. Heat map illustrating the transcript levels of top10 marker genes in each cluster of the TOE. The bars at the top of the heat map indicate the clusters in TOE.

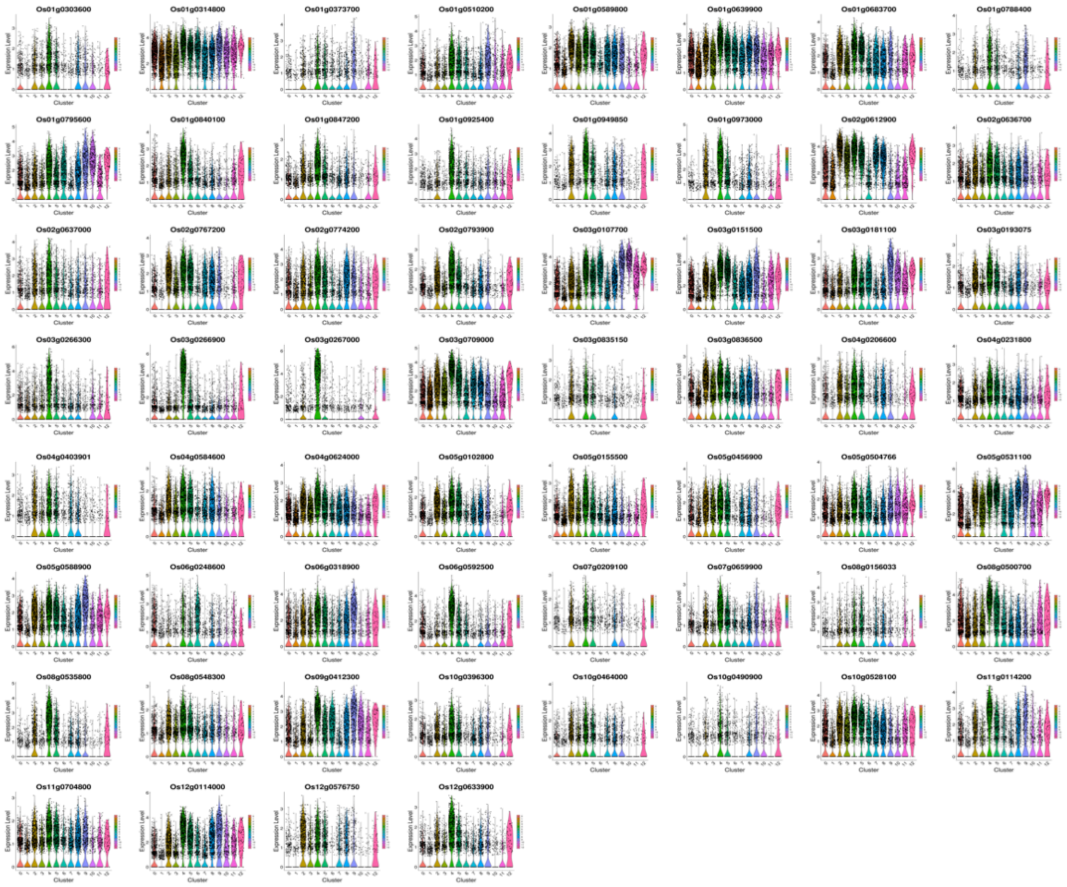

Supplementary Figure S4. Violin plot showing the expression levels of 60 of the 65 candidate genes whose expression correlated with that of OsNAC78 in the TOE. Each dot denotes a single cell.

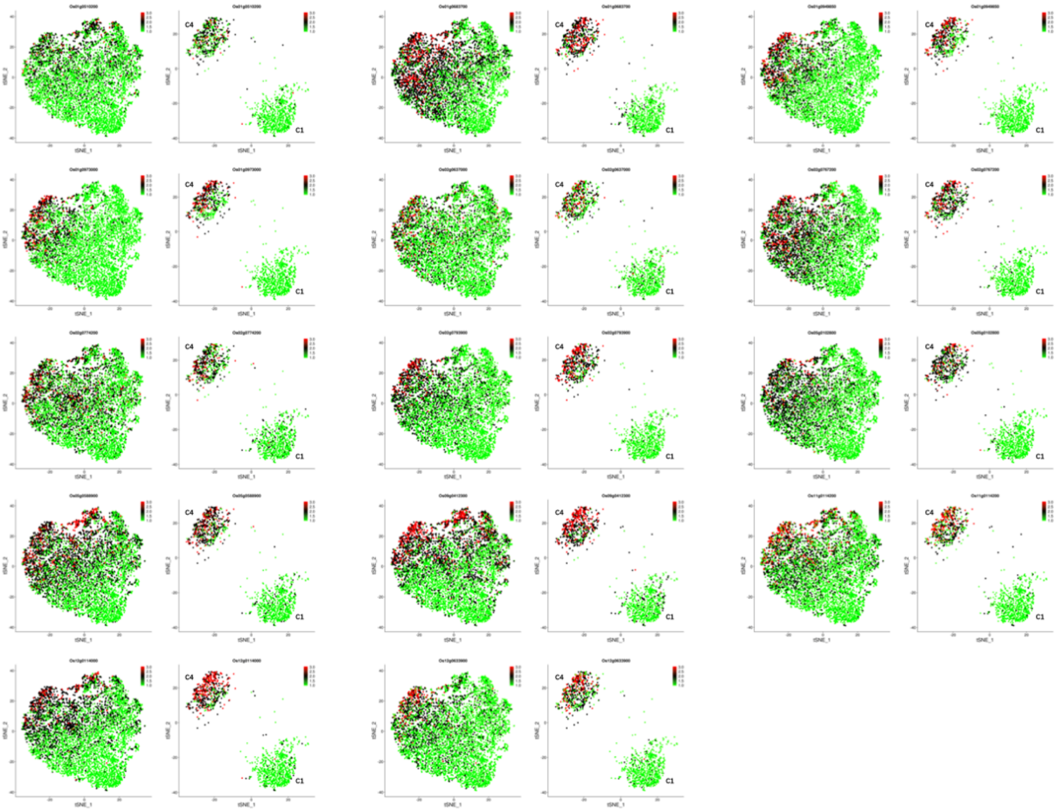

Supplementary Figure S5. t-SNE visualization of 14 of the 19 overlapping genes in the TOE and isolated from cluster 1 or 4. C1, cluster 1; C4, cluster 4. Each dot denotes a single cell. The depth of color indicates differences in expression levels.
