# Supplementary material for: Single-Cell RNA Sequencing Efficiently Predicts Transcription Factor Targets in Plants
Source: Front Plant Sci. 2020 Dec 8;11:603302. doi: 10.3389/fpls.2020.603302 (PMC7793804; doi:10.3389/fpls.2020.603302)
Supplement: Supplementary Table 5 — 65 correlation genes in the TOE. [file Data_Sheet_1.PDF]

Table S1. Primers list

Primer name	Primer sequences	Restriction enzyme sites
OsNAC78-HA-F	CAGGATATCCAGATCCAGTGGGATCC ATGAGCCAATCGCCGCCGGA	*BamH*I
OsNAC78-HA-R	ACTAGTAAGCTTGGTACCGAGCTCCC TGCTGTAGATACACAGCC	*Sac*I
OsNAC78-Q-F	AACTGGGTCATGCACGAGTA	/
OsNAC78-Q-R	TCCTCAGTCGACTCATCTGC	/
Os01g0934800-Q-F	AGGGCTCAGGAGTTGCAAT	/
Os01g0934800-Q-R	ACACCTTCTTCACCGATCCA	/
Os01g0949900-Q-F	GCTCGTTTCTGGGCTCAATT	/
Os01g0949900-Q-R	CGATGTTGGCGTACTCCTTG	/
Ubiquitin-Q-F	AACCAGCTGAGGCCCAAGA	/
Ubiquitin-Q-R	ACGATTGATTTAACCAGTCCATGA	/
Os01g0934800-F1-LacZ-F	CCGGAATTCGAAAACATCTCAATCTT CTACCT	*EcoR*I
Os01g0934800-F1-LacZ-R	CGGGGTACCCATTGCCATGTGATCCG CTC	*Kpn*I
Os01g0934800-F2-LacZ-F	CCGGAATTCGATCAATTTTATCCCGAT CT	*EcoR*I
Os01g0934800-F2-LacZ-R	CGGGGTACCCTAGATGAAAAGTTATA TTGTTT	*Kpn*I
Os01g0934800-F3-LacZ-F	CCGGAATTCTTGTCCTCTAACGTATGT CACTA	*EcoR*I
Os01g0934800-F3-LacZ-R	CGGGGTACCAGTGGGCCCAAATCTCG TGA	*Kpn*I
Os01g0934800-F4-LacZ-F	CCGGAATTCACTGTGTGTCCGTGGTGT CA	*EcoR*I
Os01g0934800-F4-LacZ-R	CGGGGTACCTTAGTGAAGATAACACG ATGTCG	*Kpn*I
Os01g0949900-F1-LacZ-F	CCGGAATTCGCTTCTCGCCAAAAACC CCA	*EcoR*I
Os01g0949900-F1-LacZ-R	CGGGGTACCGAACTAAACAGCCCCTC ATT	*Kpn*I
Os01g0949900-F2-LacZ-F	CCGGAATTCCATGAACATGTACAGTG CTC	*EcoR*I
Os01g0949900-F2-LacZ-R	CGGGGTACCCTCGCCGTCCAACCACA ATG	*Kpn*I
Os01g0949900-F3-LacZ-F	CCGGAATTCCCAAGGAGTACACCTCT GAT	*EcoR*I
Os01g0949900-F3-LacZ-R	CGGGGTACCAATAATGGTCAAAGTTG TTTTTG	*Kpn*I

Os01g0934800-LUC-F	CGGTATCGATAAGCTTAGCCCACCAGAGGTAACAA	*Hind*III
Os01g0934800-LUC-R	TTGGCGTCTTCCATGGTTTTAGCTCAGCTGCTTCGA	*Nco*I
Os01g0949900-LUC-F	CGGTATCGATAAGCTTTGGCGACCGTGCCATCTG	*Hind*III
Os01g0949900-LUC-R	TTGGCGTCTTCCATGGATTCCTGTATTCTTGGCTAAACC	*Nco*I
OsNAC78-MBP-F	GGATTTCACATATGTCCATGAGCCAATCGCCGCCGGA	*Nco*I
OsNAC78-MBP-R	ACCTGCAGGGAATTCGGATCCTCACCTGCTGTAGATACACAGCC	*BamH*I
Os01g0934800-Cy5.5-Probe1	GTGGATTTAGAGCGTGTTCGGGGGAGTT	/
Os01g0949900-Cy5.5-Probe1	GAAGGTCCCCGTGCTCCTCCATGGCGACCGTGCCATCTGC	/
Os01g0949900-Cy5.5-Probe2	GTGCTCGAGGTCATCGTGGCTGGCATTGTG	/

Table S2. The index of samples used for 10× Genomics' scRNA-seq

Sample	Cell concentration	Cell viability
Control	2080/μl	93%
TOE	1840/μl	92%

Table S3. The statistical quality results of sequencing date

Reads parameters	Reads number	
	Control	TOE
Estimated number of cells	12,891	13,639
Fraction reads in cells	89.9%	90.6%
Mean reads per cell	42,915	37,675
Median genes per cell	1,913	1,765
Median UMI counts per cell	5,116	4,696
Total genes detected	24,715	24,801
